# Supplementary material for: Meta-Analysis and Gene Set Enrichment Relative to ER Status Reveal Elevated Activity of MYC and E2F in the “Basal” Breast Cancer Subgroup
Source: PLoS One. 2009 Mar 9;4(3):e4710. doi: 10.1371/journal.pone.0004710 (PMC2650420; doi:10.1371/journal.pone.0004710)
Supplement: Dataset S1 — GSEA results from first screen. The file contains a zipped archive of the HTML results of the second GSEA screen. Reports for enriched or depleted datasets may be accessed from the “index.html” link within the my_analysis.GseaPreranked.1219975950625.rpt.zip ZIP archive. Enrichment plots are available for each of the 50 most enriched and depleted gene sets. (13.32 MB ZIP) [file pone.0004710.s001.zip › my_analysis.GseaPreranked.1219975950625.rpt.zip!//CHR14Q11.html]

Details for gene set CHR14Q11[GSEA]

|  || Dataset | zSco.ERneg.to.ERpos\_collapsed |
| Phenotype | NoPhenotypeAvailable |
| Upregulated in class | na\_neg |
| GeneSet | CHR14Q11 |
| Enrichment Score (ES) | -0.5111983 |
| Normalized Enrichment Score (NES) | -2.328294 |
| Nominal p-value | 0.0 |
| FDR q-value | 9.9529774E-5 |
| FWER p-Value | 0.0010 |
Table: GSEA Results Summary

  

Fig 1: Enrichment plot: CHR14Q11      
 Profile of the Running ES Score & Positions of GeneSet Members on the Rank Ordered List

  

| PROBE | GENE SYMBOL | GENE\_TITLE | RANK IN GENE LIST | RANK METRIC SCORE | RUNNING ES | CORE ENRICHMENT || 1 | CCNB1IP1 |  |  | 397 | 4.691 | -0.0050 | No |
| 2 | GZMB |  |  | 466 | 4.470 | 0.0137 | No |
| 3 | EFS |  |  | 1238 | 3.202 | -0.0275 | No |
| 4 | NP |  |  | 1241 | 3.199 | -0.0107 | No |
| 5 | NDRG2 |  |  | 1256 | 3.184 | 0.0053 | No |
| 6 | COCH |  |  | 1389 | 3.049 | 0.0115 | No |
| 7 | SLC39A2 |  |  | 1547 | 2.905 | 0.0151 | No |
| 8 | TGM1 |  |  | 1630 | 2.820 | 0.0240 | No |
| 9 | IL25 |  |  | 2029 | 2.507 | 0.0072 | No |
| 10 | KIAA1305 |  |  | 2475 | 2.214 | -0.0146 | No |
| 11 | STXBP6 |  |  | 3366 | 1.764 | -0.0724 | No |
| 12 | MAP4K5 |  |  | 3374 | 1.760 | -0.0636 | No |
| 13 | GZMH |  |  | 3482 | 1.717 | -0.0625 | No |
| 14 | SLC7A7 |  |  | 3876 | 1.563 | -0.0839 | No |
| 15 | FAM12A |  |  | 3983 | 1.517 | -0.0838 | No |
| 16 | TRA@ |  |  | 4156 | 1.450 | -0.0891 | No |
| 17 | MGC40069 |  |  | 4536 | 1.307 | -0.1108 | No |
| 18 | MMP14 |  |  | 5099 | 1.116 | -0.1473 | No |
| 19 | CPNE6 |  |  | 5126 | 1.108 | -0.1434 | No |
| 20 | TRAC |  |  | 5277 | 1.057 | -0.1491 | No |
| 21 | MYH7 |  |  | 5304 | 1.044 | -0.1455 | No |
| 22 | TEP1 |  |  | 5387 | 1.018 | -0.1462 | No |
| 23 | RNASE1 |  |  | 5702 | 0.910 | -0.1651 | No |
| 24 | LTB4R2 |  |  | 5899 | 0.847 | -0.1754 | No |
| 25 | AP1G2 |  |  | 6018 | 0.805 | -0.1801 | No |
| 26 | CEBPE |  |  | 6128 | 0.775 | -0.1842 | No |
| 27 | PABPN1 |  |  | 6462 | 0.664 | -0.2058 | No |
| 28 | NGDN |  |  | 7257 | 0.399 | -0.2637 | No |
| 29 | SLC22A17 |  |  | 7406 | 0.344 | -0.2730 | No |
| 30 | PRKD1 |  |  | 7686 | 0.236 | -0.2928 | No |
| 31 | PYGL |  |  | 7720 | 0.223 | -0.2941 | No |
| 32 | PARP2 |  |  | 7734 | 0.219 | -0.2939 | No |
| 33 | APEX1 |  |  | 7763 | 0.205 | -0.2950 | No |
| 34 | MYH6 |  |  | 7835 | 0.175 | -0.2994 | No |
| 35 | REC8L1 |  |  | 7917 | 0.140 | -0.3048 | No |
| 36 | FAM12B |  |  | 8185 | 0.044 | -0.3247 | No |
| 37 | RPGRIP1 |  |  | 8569 | -0.110 | -0.3531 | No |
| 38 | NRL |  |  | 8671 | -0.158 | -0.3599 | No |
| 39 | MRPL52 |  |  | 8770 | -0.196 | -0.3662 | No |
| 40 | PSME2 |  |  | 8865 | -0.240 | -0.3721 | No |
| 41 | CMA1 |  |  | 8878 | -0.246 | -0.3716 | No |
| 42 | PSMB5 |  |  | 9055 | -0.339 | -0.3831 | No |
| 43 | ZNF409 |  |  | 9232 | -0.419 | -0.3942 | No |
| 44 | HNRPC |  |  | 9598 | -0.616 | -0.4185 | No |
| 45 | SUPT16H |  |  | 9683 | -0.660 | -0.4213 | No |
| 46 | LTB4R |  |  | 9787 | -0.716 | -0.4253 | No |
| 47 | ZNF219 |  |  | 9805 | -0.727 | -0.4227 | No |
| 48 | RNASE6 |  |  | 10023 | -0.858 | -0.4345 | No |
| 49 | ARHGAP5 |  |  | 10590 | -1.235 | -0.4707 | No |
| 50 | ACIN1 |  |  | 10688 | -1.310 | -0.4711 | No |
| 51 | METTL3 |  |  | 10984 | -1.554 | -0.4851 | No |
| 52 | ISGF3G |  |  | 11042 | -1.610 | -0.4808 | No |
| 53 | C14ORF93 |  |  | 11445 | -1.987 | -0.5006 | Yes |
| 54 | C14ORF122 |  |  | 11562 | -2.097 | -0.4982 | Yes |
| 55 | NFATC4 |  |  | 11581 | -2.108 | -0.4883 | Yes |
| 56 | PRMT5 |  |  | 11587 | -2.113 | -0.4775 | Yes |
| 57 | FLJ10357 |  |  | 11723 | -2.244 | -0.4757 | Yes |
| 58 | ABHD4 |  |  | 11877 | -2.411 | -0.4744 | Yes |
| 59 | RNF31 |  |  | 11884 | -2.421 | -0.4620 | Yes |
| 60 | RBM23 |  |  | 11919 | -2.455 | -0.4515 | Yes |
| 61 | C14ORF92 |  |  | 11944 | -2.490 | -0.4400 | Yes |
| 62 | PSME1 |  |  | 12047 | -2.633 | -0.4337 | Yes |
| 63 | WDR23 |  |  | 12215 | -2.861 | -0.4311 | Yes |
| 64 | DHRS2 |  |  | 12228 | -2.884 | -0.4166 | Yes |
| 65 | DAD1 |  |  | 12352 | -3.025 | -0.4098 | Yes |
| 66 | BCL2L2 |  |  | 12432 | -3.152 | -0.3990 | Yes |
| 67 | C14ORF94 |  |  | 12442 | -3.168 | -0.3828 | Yes |
| 68 | METT11D1 |  |  | 12459 | -3.196 | -0.3670 | Yes |
| 69 | RABGGTA |  |  | 12503 | -3.266 | -0.3529 | Yes |
| 70 | KIAA0323 |  |  | 12674 | -3.567 | -0.3467 | Yes |
| 71 | DHRS4 |  |  | 12679 | -3.574 | -0.3280 | Yes |
| 72 | NEDD8 |  |  | 12714 | -3.645 | -0.3111 | Yes |
| 73 | OXA1L |  |  | 12740 | -3.716 | -0.2932 | Yes |
| 74 | DHRS1 |  |  | 12757 | -3.763 | -0.2744 | Yes |
| 75 | IPO4 |  |  | 12777 | -3.812 | -0.2555 | Yes |
| 76 | SALL2 |  |  | 12781 | -3.823 | -0.2354 | Yes |
| 77 | CHMP4A |  |  | 12863 | -4.045 | -0.2200 | Yes |
| 78 | PIGH |  |  | 12912 | -4.186 | -0.2013 | Yes |
| 79 | TINF2 |  |  | 12919 | -4.211 | -0.1793 | Yes |
| 80 | SLC7A8 |  |  | 12952 | -4.333 | -0.1587 | Yes |
| 81 | CHD8 |  |  | 13000 | -4.454 | -0.1385 | Yes |
| 82 | THTPA |  |  | 13092 | -4.796 | -0.1198 | Yes |
| 83 | RNASE4 |  |  | 13230 | -5.708 | -0.0998 | Yes |
| 84 | TM9SF1 |  |  | 13246 | -5.882 | -0.0696 | Yes |
| 85 | GMPR2 |  |  | 13300 | -6.637 | -0.0382 | Yes |
| 86 | LRP10 |  |  | 13314 | -7.445 | 0.0005 | Yes |
Table: GSEA details [plain text format]

  

Fig 2: CHR14Q11: Random ES distribution      
 Gene set null distribution of ES for **CHR14Q11**
